# Structural and Adsorption Properties of ZIF-8-7 Hybrid Materials Synthesized by Acid Gas-Assisted and *De Novo* Routes

**DOI:** 10.1021/acs.jpcc.3c06334

**Published:** 2023-11-30

**Authors:** Arvind Ganesan, Peter C. Metz, Raghuram Thyagarajan, Yuchen Chang, Stephen C. Purdy, Krishna C. Jayachandrababu, Katharine Page, David S. Sholl, Sankar Nair

**Affiliations:** † School of Chemical & Biomolecular Engineering, Georgia Institute of Technology, Atlanta, Georgia 30332, United States; ‡ Materials Science and Engineering Department, University of Tennessee, Knoxville, Tennessee 37996, United States; § Neutron Scattering Division, Oak Ridge National Laboratory, Oak Ridge, Tennessee 37830, United States; ∥ Oak Ridge National Laboratory, Oak Ridge, Tennessee 37830, United States

## Abstract

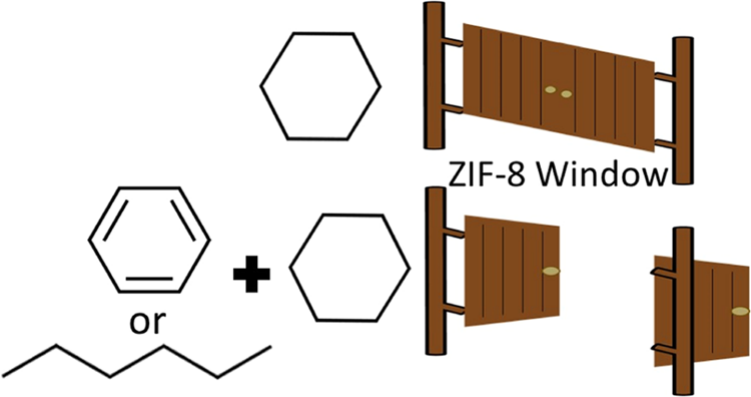

The tuning of micropore environments in zeolitic imidazolate frameworks (ZIFs) by mixed-linker synthesis has the potential for enabling new molecular separation properties. However, *de novo* synthesis of mixed-linker (hybrid) ZIFs is often challenging due to the disparate chemical properties of the different linkers. Here, we elucidate the structure and properties of an unconventional ZIF-8-7 hybrid material synthesized via a controlled-acid-gas-assisted degradation and reconstruction (solvent-assisted crystal redemption, SACRed) strategy. Selective insertion of benzimidazole (ZIF-7 linker) into ZIF-8 using SACRed is used as a facile method to generate a ZIF-8-7 hybrid material that is otherwise difficult to synthesize by *de novo* methods. Detailed crystal structure and textural characterizations clarify the significant differences in the microstructure of the SACRed-derived ZIF-8-7 hybrid material relative to a *de novo* synthesized hybrid of the same overall linker composition as well as the parent ZIF-8 material. Unary and binary adsorption measurements reveal the tunability of adsorption characteristics as well as the prevalence of nonideal cooperative mixture adsorption effects that lead to large deviations from predictions made with ideal adsorbed solution theory.

## Introduction

Zeolitic imidazole frameworks such as ZIF-8 are of interest for potential use in hydrocarbon separations.^1−5^ Their high crystallinity, stability, hydrophobicity, and linker functionalities provide separation mechanisms based on selective adsorption and diffusion. Although ZIF-8 has a pore window with a nominal crystallographic size of 3.4 Å, significant adsorption of molecules larger than the ZIF-8 pore window has been observed due to the high flexibility of the framework.^3−7^ Recent efforts have also studied the effect of flexibility and linker motion in ZIF-8 with solid-state NMR.^4,8−10^ ZIF-8 has shown impressive functionality in biofuel separation,^6,11^ benzene/cyclohexane separation,^3^ and separation of xylene isomers.^1^ The ability to introduce more than one linker in the zeolitic imidazolate framework (ZIF) framework has been shown to enable tuning of separation performance while maintaining the advantages of the original parent/template ZIF.^12−20^ In the case of ZIFs, these initial efforts involved the use of imidazolate linkers that could easily cocrystallize together with the Zn metal centers. Many imidazolate linkers with bulky functional substituents (such as aromatic groups) could enable new separation properties, but these linkers are considerably more difficult to cocrystallize *de novo* with simpler linkers like 2-methylimidazole (2-MeIm, the ZIF-8 linker) due to differences in solubility, deprotonation equilibria, and steric effects. The simplest such linker with an aromatic substituent is benzimidazole (BzIm) which forms ZIF-7. Our early *de novo* synthesis efforts showed only limited ability to incorporate BzIm into ZIF-8.^14,21^ However, our later development of SACRed allowed facile incorporation of BzIm linkers into the ZIF-8 structure with variable BzIm:2-MeIm linker ratios.^14,21^ In this method, humid SO_2_ was used to degrade ZIF-8 by controlled demolition of the Zn-linker coordination bonds, thereby allowing the 2-MeIm linkers to be more easily replaced by the introduction of BzIm linkers to create a ZIF-8-7 hybrid material preserving the original structural topology (SOD) of the ZIF-8 “template” material. In addition to the insertion of fresh linkers, the SACRed treatment recovers/heals molecular defects caused during the acid gas exposure.^22,23^

ZIF-8-7 hybrid materials have been considered for CO_2_/CH_4_ separation.^24^ In another example, postsynthetic insertion of 5,6-dimethylbenzimidazole^25^ in ZIF-8 enabled excellent C_2_H_4_/C_2_H_6_ and C_3_H_6_/C_3_H_8_ separation performance. Some of these separations are hypothesized to occur due to changes in the effective pore size or surface functionality. Others are hypothesized to be related to changes in rigidity/flexibility of the hybrid ZIF framework relative to ZIF-8. For example, control of ZIF-8 framework flexibility using current-driven synthesis^26^ and electric field-induced libration control^27^ have also been shown to enable more selective separation of C_3_H_6_/C_3_H_8_ mixtures.^28^ Following the initial demonstration of ZIF-8-7 hybrid material synthesis using SACRed,^21^ this technique was more recently extended to reconstruction of other MOFs such as ZIF-71, ZIF-90, UiO-66, and UiO-67.^22^

If mixed-linker ZIFs can enable efficient separation of C_1_–C_3_ molecules, then the separation of larger molecules (such as C_6_ hydrocarbon mixtures in this study) could also potentially be tunable. Here, we report a detailed structural and functional characterization study of ZIF-8-7 hybrid materials, with particular focus on comparing the materials synthesized by SACRed and conventional *de novo* strategies. The structural and textural differences between the ZIF-8-7 hybrids are investigated with X-ray diffraction and N_2_ physisorption. The adsorption properties are probed by measuring the C_6_ hydrocarbon (*n*-hexane, benzene, and cyclohexane) unary and binary adsorption characteristics of three materials: ZIF-8, ZIF-8-7_*de novo*, and ZIF-8-7_SACRed. We find that the adsorption and mixture separation behavior can be modified by changes in adsorbent–adsorbate interactions, and additionally, the two types of hybrid ZIF-8-7 materials show significantly different adsorption behavior. Hence, this work introduces the concept of synthesizing mixed-linker/hybrid ZIFs that are structurally and functionally different while having the same/similar linker composition.

## Experimental Methods

### Materials

Zinc nitrate hexahydrate (Sigma-Aldrich), benzimidazole (BzIm) (Sigma-Aldrich), 2-methylimidazole (2-MeIm) (Sigma-Aldrich), methanol (VWR), benzene (Sigma-Aldrich), hexane (Sigma-Aldrich), cyclohexane (Sigma-Aldrich), chloroform-d (CDCl_3_) (Millipore Sigma), 2-propanol (IPA) (VWR), 1,3,5-triisopropylbenzene (TIPB) (Sigma-Aldrich), deuterated acetic acid (CD_3_COOD), and ethanol (VWR) were used as received. Deionized water from an EMD Millipore water purification system, 1000 ppm SO_2_ balance N_2_, ultrahigh purity hydrogen, helium, and air (76.5–80.5% N_2_, 19.5–23.5% O_2_) from Airgas were used in this work.

### *De Novo* ZIF Synthesis

ZIF-8 was synthesized by the procedure given by Jian et al.^29^ First, 0.744 g of zinc nitrate was dissolved in 10 mL of deionized (DI) water. 10.25 g of 2-MeIm was dissolved in 90 mL of DI water. The metal salt solution was added to the linker solution. The solution was stirred at 200 rpm at room temperature for 24 h. The crystals were centrifuged for 10 min at 8500 rpm and washed with fresh methanol. The crystals were degassed at 453 K in vacuum overnight. ZIF-8-7_*de novo* was synthesized with a procedure modified from Thompson et al.^14^ Typically, 0.228 g of BzIm and 3.122 g of 2-MeIm were dissolved in 60 mL of MeOH. 1.485 g of zinc nitrate was dissolved in 60 mL of MeOH. The metal solution was stirred with the linker solution for 1 h at room temperature. The crystals were centrifuged for 10 min at 8500 rpm and washed with fresh methanol. The crystals were degassed at 453 K in vacuum overnight.

### SACRed ZIF Synthesis

Acid gas exposures were performed in a custom-built system described in our earlier work.^22^ Degassed MOF samples were exposed to a preset 100 ppm-days (concentration × time) of SO_2_ at 85% relative humidity (RH) to introduce the controlled degradation in ZIF-8. 1000 ppm SO_2_ in N_2_ lecture bottles were used as the acid gas source. Humidity was generated with a relative humidity (RH) generator (Fuel Cell Technologies Inc.). A safe working environment was ensured with two Dräger PAC 7000 SO_2_ personal monitors (inside and outside the fume hood). The insertion of BzIm into the acid gas-exposed ZIF-8 material was performed with a 0.25 M linker solution in methanol. Around 150 mg of degraded ZIF-8 sample was dispersed in 40 mL of BzIm solution inside Teflon-lined Parr autoclaves. The autoclaves were incubated for 48 h at 363 K without any rotation. The recovered crystals (ZIF-8-7_SACRed) were centrifuged from the mother liquor and rinsed three times with fresh methanol. Overnight vacuum degassing of the recovered crystals was performed at 453 K.

### Characterization

Physical and chemical characterization of activated ZIF samples (pristine, acid gas-exposed, *de novo*, and SACRed) were performed. Powder X-ray diffraction (PXRD) patterns were obtained in an X’Pert Pro PANalytical diffractometer (Cu Kα source, λ = 0.1541 nm). The Cu anode was operated at 45 kV and 40 mA for collecting PXRD patterns with a scan time of 12 steps/s and a step size of 0.0167° 2θ over the range of 5–50° 2θ. Micromeritics Tristar II 3020 and MicroActive V3 analyzers were used to obtain the Brunauer–Emmett–Teller (BET) surface area and t-plot pore volume from N_2_ adsorption isotherms at 77 K. BET parameters were optimized for each N_2_ isotherm using the Rouquerol criteria.^30^ SEM images were obtained with a Hitachi SU 8010 scanning electron microscope. Organics were analyzed with a Shimadzu GC 2010 instrument with IPA as the solvent. Solution NMR measurements of organics were performed with a Bruker Avance III 400 MHz with CDCl_3_ as the solvent. Solution NMR measurements of digested ZIF samples were performed with CD_3_COOD as the solvent in a Bruker Avance III 400 MHz. Single component vapor sorption isotherms at 30 °C were obtained using a dynamic vapor sorption device DVS-Advantage (Surface Measurement Systems). All samples were degassed for 6 h under nitrogen flow at 100 °C. The degassed samples were exposed to a flowing stream of organic vapor at various partial pressures between 0 and 95%. X-ray diffraction measurements were performed at beamline 11-ID-B of the Advanced Photon Source (APS) at Argonne National Laboratory using a monochromated 0.2113 Å (58.677 keV) beam. ZIF-8, ZIF-8-7_*de novo*, and ZIF-8-7 SACRed samples (with ∼25% benzimidazole substitution in *de novo* and SACRed) from Jayachandrababu et al.^21^ were activated in a vacuum oven at 363 K for 3 h, packed into 1 mm OD polyimide capillaries, and sealed at both ends with epoxy. Two-dimensional (2D) powder diffraction intensities were measured using a PerkinElmer large area detector with a sample-to-detector distance of 1000 mm. Detector geometry was calibrated by using a CeO_2_ standard. Pattern calibration, integration, and experimental background subtraction were performed using the software package DIOPTAS.^31^ Rietveld refinement was performed using the software package Topas 7.^32^ Rigid bodies were defined for the organic molecules, reducing the parameter space to a few bond angles and distances, two Euler angles, and three centroid coordinates. The software package ISODISTORT was employed to identify likely symmetry subgroups in the benzimidazole-substituted specimens and to identify likely symmetry mode distortions constraining the framework site displacements.^33^

### Adsorption Measurements and Modeling

For batch adsorption measurements, typically, 100 mg of ZIF-8 was dispersed in 5 mL of TIPB. One mmol of benzene + cyclohexane each, hexane + cyclohexane each, hexane + benzene each, and 1 mmol of cyclohexane were added to three different MOF dispersions. The mixture was stirred at 200 rpm for 6 h. The supernatant was filtered with a 0.2 μm poly(tetrafluoroethylene) (PTFE) filter and analyzed with a Shimadzu GC 2010 Plus.

For breakthrough adsorption column preparation, ZIF pellets were prepared in a pellet press die set without a binder. Large pellets were pressed in the die for 60 s at 1000 psi. These were ground and sieved to maintain a particle size between 425 and 600 μm. The particles were packed into a 50 mm stainless-steel column with a diameter of 0.46 mm. Frits were added to both ends to prevent the loss of adsorbent particles. Multicomponent liquid adsorption data were collected with a liquid breakthrough system. The measurement and analysis procedures were obtained from previous work in our research group.^34^ The ZIF-packed columns were reactivated at 383 K under vacuum for 24 h prior to breakthrough runs, followed by feeding the desorbent (ethanol) liquid at room temperature for 60 min. The separation performance of ZIF-8 and ZI-8-7 hybrids was tested for equimolar hexane/benzene and benzene/cyclohexane binary mixtures at room temperature. TIPB was used as a tracer to determine the dead volume of the packed bed. The feed and desorbent flows were always maintained at 0.2 mL/min. 0.2 mL of sample was collected for each time point. Organic composition analysis for the hexane/benzene and benzene/cyclohexane mixtures was performed with Shimadzu GC 2010 Plus and solution NMR, respectively. The adsorbed amounts of species *i* (*q*_*i*_, mmol/g) were estimated as follows:
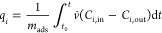
1

Here, *m*_ads_ is the MOF loading in the column (g),  is the feed flow rate (cm^3^/min), *C*_*i*,in_ and *C*_*i*,out_ are the concentrations of the species *i* at the column inlet and outlet, respectively (mol/cm^3^), and *t* is the time (min). The adsorption (liquid–solid equilibrium) separation factor is defined as
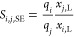
2where *x*_*i*,L_ and *x*_*j*,L_ are the mole fractions of species *i* and *j* of the liquid feed mixture, whereas q_*i*_, and *q*_*j*_ are the adsorption uptakes. For ideal adsorbed solution theory (IAST) predictions, the adsorbate vapor pressures were calculated using Raoult’s law (***xP***_***i*****,sat**_ = ***y***_***i***_***P***), where *x*_*i*_ is the mole fraction of *i* in the liquid phase, *P*_*i*,sat_ is the saturation pressure of component *i*, *y*_*i*_ is the mole fraction in the vapor phase, and *P* is the total pressure. Binary IAST adsorption calculations were performed for two equimolar binary mixtures, hexane/benzene and benzene/cyclohexane.^35,36^ IAST is a commonly used method to predict mixture adsorption isotherms from unary adsorption isotherm data in nanoporous materials.^37,38^ The single-component vapor-phase adsorption isotherms for hexane, benzene, and cyclohexane in the three ZIF materials were fit to the best-fit models (Langmuir, Dual-site Langmuir, Langmuir–Freundlich, etc.) using IAST++ software.^39^

### Computational Methods

ZIF-7 and ZIF-11 structure models were obtained from the work by Park and co-workers.^40,41^ These structures were optimized using first-principles density functional theory (DFT) calculations as implemented in VASP software.^42,43^ These calculations used the projected augmented-wave pseudopotentials^44,45^ with the generalized gradient approximation using the Perdew–Burke–Ernzerhof (PBE) functional.^46^ We used a plane-wave cutoff energy of 520 eV with an energy tolerance of 10^–4^ eV for electronic minimization and 0.03 eV for ionic minimization self-consistent cycles. Since the unit cell dimensions were large, the reciprocal space was sampled only at the Γ point.

## Results and Discussion

**Figure 1:**
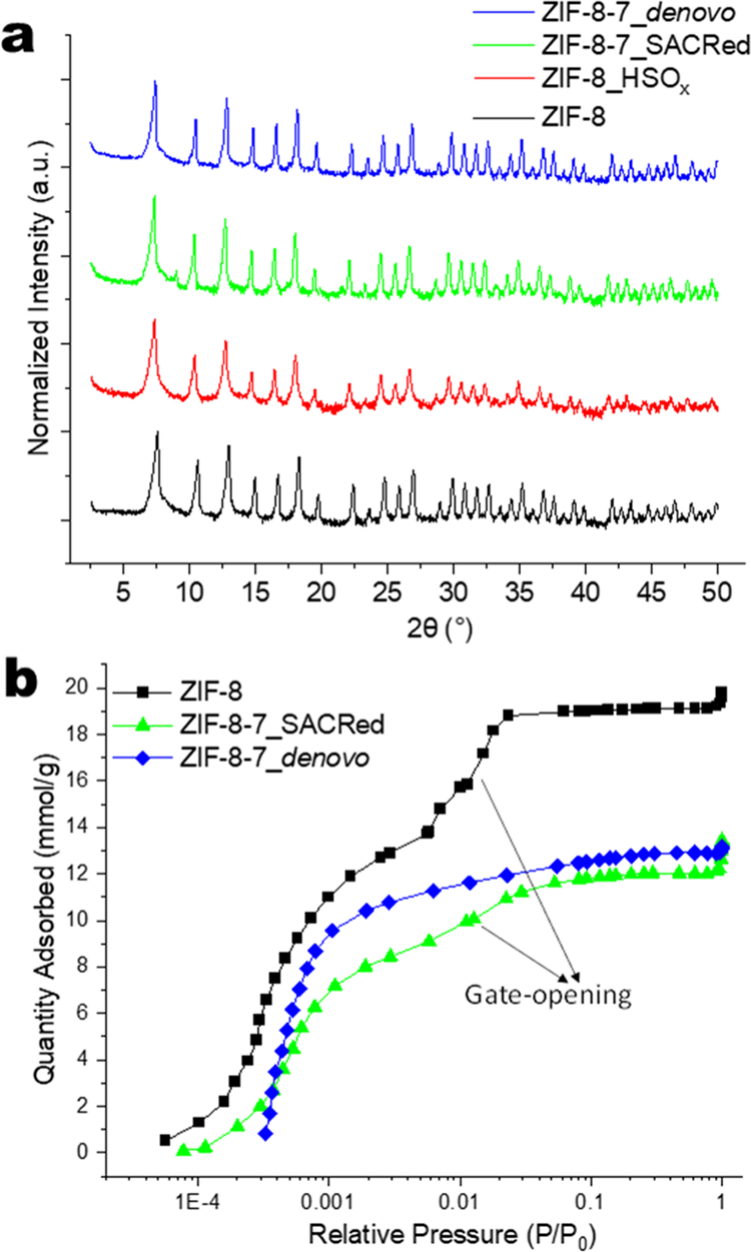
(a) Powder XRD patterns and (b) N_2_ adsorption isotherms of the four materials: ZIF-8, ZIF-8_HSO_*x*_, ZIF-8-7_SACRed, and ZIF-8-7_*de novo*.

The crystallinity of four materials, pristine ZIF-8, ZIF-8 after exposure to acid gas (ZIF-8_HSO_*x*_), ZIF-8-7_SACRed, and ZIF-8-7_*de novo*, is characterized by PXRD (Figure 1a). The patterns are normalized with respect to the most intense Bragg peak and plotted on a log scale with a vertical stacking offset. Both ZIF-8-7_*de novo* and ZIF-8-7_SACRed have the same overall linker composition (20% BzIm and the remaining 80% 2-MeIm), as determined by ^1^H solution NMR of the digested materials (Figure S1). In the acid gas-degraded ZIF-8_HSO_*x*_, a significant loss of peak intensity corresponding to short-range order (>20° 2θ) is observed relative to the pristine ZIF-8 (due to breakage of Zn–N coordination bonds and formation of acid gas–linker defect complexes^26^), but the retention of characteristic longer-range order peaks (<20° 2θ) signifies that the structure retains the overall SOD topology of ZIF-8. The reappearance of short-range order peaks with the incorporation of BzIm linkers in the ZIF-8-7_SACRed material confirms the reconstruction of the ZIF material in the form of a ZIF-8-7 hybrid. This is similar to our earlier observations confirming the introduction of disorder with acid gas exposure and recovery of crystallinity with non-native linker insertion.^21^ The PXRD pattern of the ZIF-8-7_*de novo* material indicates that it has the same SOD topology of ZIF-8.

Figure 1b compares the N_2_ physisorption isotherms of the four materials at 77 K. The characteristic “gate-opening” behavior is seen in ZIF-8,^6,28,47^ as indicated by the inflection around *P*/*P*_0_ = 0.01. This same effect is observed in ZIF-8_HSO_*x*_ and ZIF-8-7_SACRed. This behavior is in direct contrast with ZIF-8-7_*de novo*, which shows a more rigid pore structure with no gate-opening inflection, even though the two hybrid materials have similar overall linker compositions (20% BzIm/80% 2-MeIm). Table S1 (Supporting Information) shows the BET surface area and the *t*-plot micropore volume obtained from the isotherms. Acid gas exposure leads to a 45% loss of pore volume and BET surface area. With BzIm insertion, the degree of recovery of porosity and surface area is attributed to both the reconstruction of crystallinity and substitution of 2-MeIm linkers with bulkier BzIm linkers. The BET surface area and pore volume of ZIF-8-7_*de novo* are also similar to that of the SACRed analogue, enabling direct comparison of their properties. In summary, Figure 1 indicates both structural differences and similarities of the two hybrid materials.

**Figure 2:**
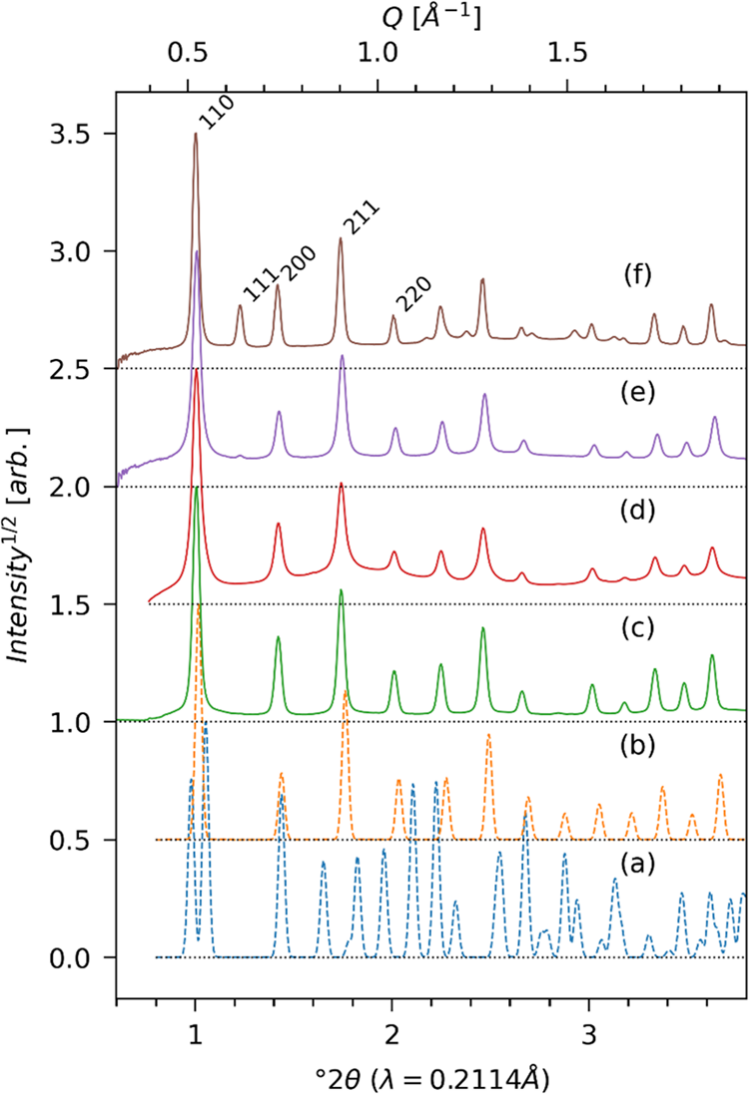
Calculated profiles for (a) ZIF-7 and (b) ZIF-8 and measured profiles for (c) ZIF-8, (d) ZIF-8-HSO_*x*_, (e) ZIF-7-8_*de novo*, and (f) ZIF-7-8_SACRed. Note the emergence of the 111 Bragg reflection in parts e and f, indicating the loss of I lattice centering.

Figure 2 shows synchrotron X-ray diffraction data from the four materials. The data are normalized with respect to the most intense Bragg peak and plotted on a square root intensity scale with vertical stacking offsets. Pristine ZIF-8 (Figure 2c) shows both the sharp Bragg peaks corresponding to the simulated ideal framework structure of ZIF-8 (Figure 2b) as well as weak diffuse features associated with framework disorder.^22,23,48,49^ The diffuse intensity increases following acid gas exposure (Figure 2d) proportionate to the concentration of linker defects,^48,49^ and the Bragg peak width increases due to the shortened coherent domain size and increased microstrain. Upon BzIm substitution in ZIF-8-7_*de novo* (Figure 2e) and ZIF-8-7_SACRed (Figure 2f), the Bragg peaks are sharpened again, indicating increased coherency in their framework structures. However, the clear emergence of the 111 Bragg reflection in ZIF-8-7_SACRed clearly indicates a loss of I-centering (i.e., systematic absence of *h* + *k* + *l* ≠ 2*n* reflections) and a lower-symmetry structure. This 111 reflection is only weakly observed in ZIF-8-7_*de novo* (Figure 2e), which appears to be much closer to the pristine ZIF-8 structure. Finally, it is clear that the two ZIF-8-7 hybrid materials cannot be described as simple physical mixtures of the two parent/end-member structures (ZIF-8 and ZIF-7 simulated ideal patterns shown in Figure 2a,b).

There are many possible strategies for indexing powder patterns. In the present case, the synthesis methods suggest that the product ZIF-8-7 phases can be described by a symmetry subgroup of the parent structure. It is anticipated that the parent structure must distort to accommodate the substituted linker, which contains a relatively bulky benzene group. Thus, we explored candidate structures using the ISODISTORT software package^33^ to elucidate plausible symmetry subgroups and the symmetry mode distortions of the linkers and Zn sites driving the lattice deformation. As input, we reduced the crystal structure to two characteristic sites: the Zn metal center and the centroid of the imidazolate ring, allowing for displacement modes in both cases as well as rotational modes for the linker. Candidate structures with minimal cell volume and degrees of freedom were sought, giving preference to derived lattices with null propagation vectors, i.e., *k* = (0, 0, 0), and the highest possible symmetry. Symmetry subgroups of the nominal I-centered cubic lattice only produced the systematically absent reflection for noninteger propagation vectors, giving rise to superlattices of 2–8 times the volume of the parent cell. Consequently, we decreased the input symmetry to *P*23. Trial cells and their symmetry-constrained strain modes were tested against the data by Le Bail refinement in Tops v7.^32^ While the elimination of the I-lattice centering was able to reproduce the primitive cubic 111 reflections, the additional weak features present in the ZIF-8-7_SACRed synchrotron XRD pattern could not be reproduced by any symmetry-constrained cubic, trigonal, tetragonal, or orthorhombic setting. Thus, we infer that the SACRed ZIF-8-7 is pseudocubic with SOD (sodalite) topology but with monoclinic symmetry at most due to significant structural distortions. On the other hand, the ZIF-8-7*_de novo* material is adequately described by a *P*23 pace group setting.

**Figure 3:**
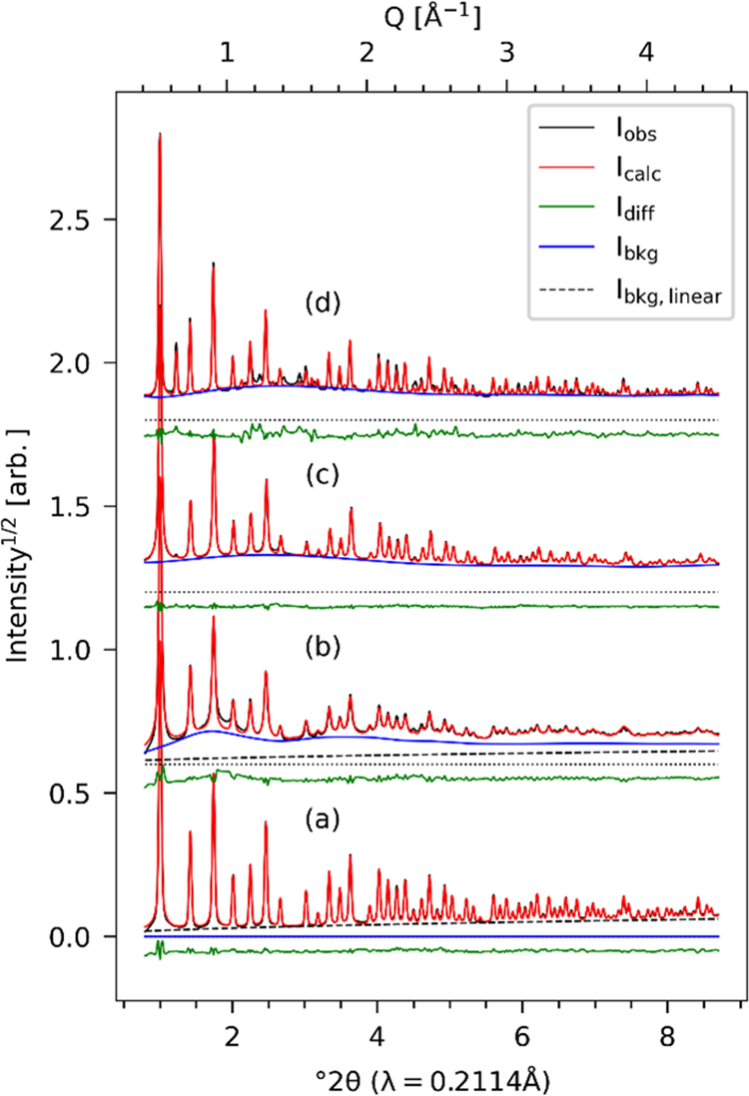
Rietveld refinements for (a) ZIF-8, (b) ZIF-8-HSO_*x*_, (c) ZIF-8-7_*de novo*, and (d) ZIF-8-7_SACRed. Key refined parameters are given in Table 1.

Rietveld refinements of these patterns were attempted using rigid-body descriptions of the organic linker molecules (as implemented in Topas v7) and symmetry mode distortions of the framework identified by ISODISTORT (Figure 3). We have recently reported similar refinements for pristine and partially disordered ZIF-8 using amorphous profiles derived from severely degraded analogous materials.^49^ In these cases, the background intensity is fitted as a combination of a linear background and the scaled amorphous profile. Refinement of the pristine ZIF-8 pattern (Figure 3a) converged easily with no constraint on the Euler angles describing the linker orientation. Refinement of the partially degraded ZIF-8_HSO_X_ pattern (Figure 3b) required addition of an amorphous profile as previously described.^49^ For the two BzIm-substituted hybrid materials, the background intensity was fitted by a Chebyshev polynomial. The ZIF-8-7_*de novo* pattern was also easily fit by rigid-body Rietveld refinement assuming a solid solution of the two linker types and employing the cubic *P*23 cell and symmetry modes identified by ISODISTORT (Figure 3c). However, the Rietveld refinement of a physically realistic model for the ZIF-8-7_SACRed material in a P2 space group setting proved intractable. Therefore, the final fitting of the ZIF-8-7_SACRed pattern using a similar cubic *P*23 cell thus omits several weak reflections and should be viewed as an oversymmetrized model of the real structure (Figure 3d).

Structure information derived from the analysis presented above is presented in Table 1, including the fitted lattice parameters (*a*), the estimated density (ρ), the coherent domain size (*D*_L_), and the microstrain (ε). As previously reported,^49^ the refined microstrain value for the acid gas-exposed sample increases markedly in response to dangling linker formation in the sodalite framework. We see a reduction of microstrain in the crystal structure after benzimidazole substitution, implying that the linker substitution repairs the framework connectivity. We also analyzed another measure, *∑*, the mean-square symmetry mode amplitude, defined by
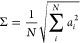
3

**Table 1:** Key Structural Parameters Were Obtained by Rietveld Refinement^a^

material	symmetry group	*a* [Å]	*c* [Å]	ρ [g cm^–3^]	*D*_L_ [Å]	ε × 10^–6^	∑	*R*_wp_ [%]
Reported^41,50^								
ZIF-8	*I*43̅*m*	17.0095(8)		0.921				
ZIF-7^b^	*R*3̅	22.989(3)	15.763(3)	1.390				
This Work								
ZIF-8	*I*23	17.0270(3)		1.0048(7)	520(30)	500(18)	0.07(3)	8.31
ZIF-8_HSO_*x*_	*I*23	17.028(2)		0.9349(3)	600(150)	2860(10)	0.08(1)	10.3
ZIF-8-7_SACRed^c^	*P*23	17.0334(8)		0.9927(1)	356(28)	210(40)	0.8(4)	11.0
ZIF-8-7*_de novo*	*P*23	16.9549(4)		1.0238(7)	115(1)	690(20)	0.20(7)	3.76

a All data were obtained at 300 K except where noted.

b 258 K.

c Could not be fully indexed, treated as pseudocubic.

A value of ∑ = 0 indicates a framework with Zn and imidazolate centroid positions undistorted from the reported ZIF-8 structure. Values for this measure for each material are given in Table 1. By this measure, we find a second indication that the SACRed and *de novo* synthesis routes yield subtly different products. The ZIF-8-7_SACRed material appears to have a larger structural distortion than the ideal ZIF-8 SOD framework.

**Figure 4:**
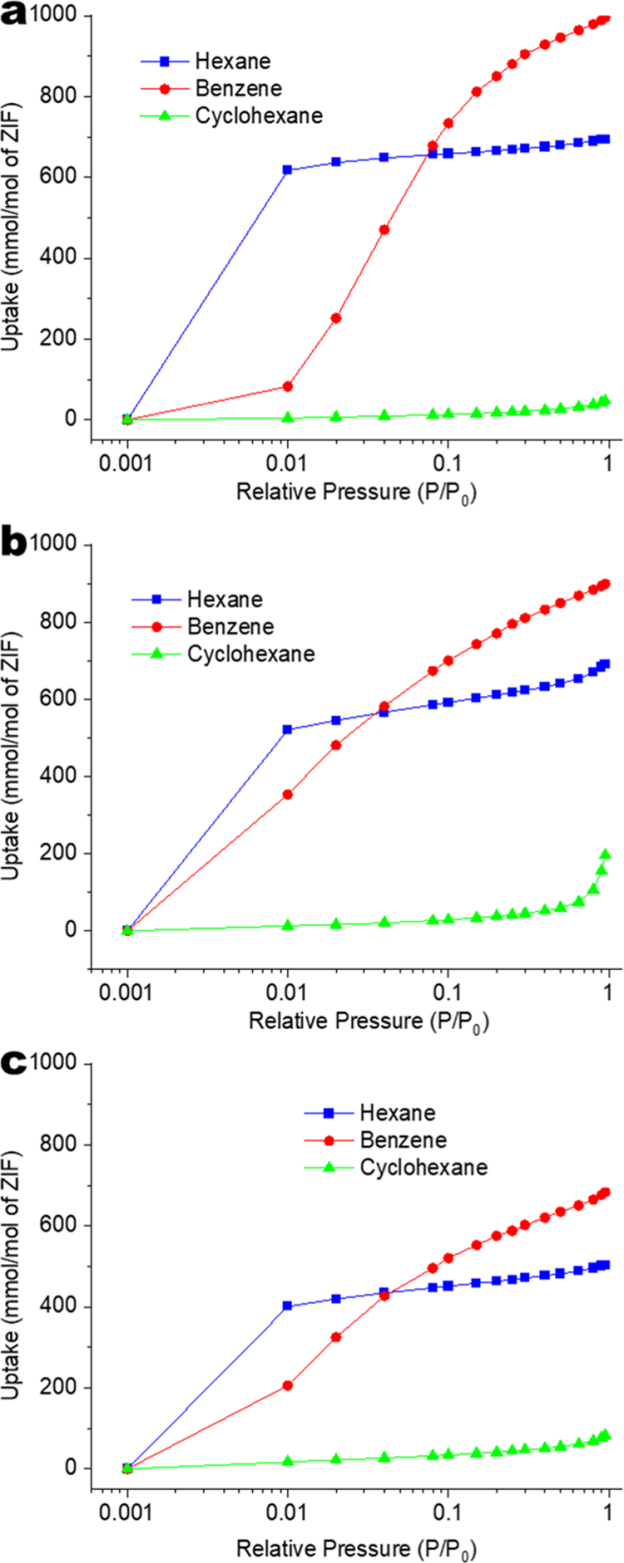
Single-component vapor adsorption isotherms of (a) ZIF-8, (b) ZIF-8-7_*de novo*, and (c) ZIF-8-7_SACRed.

Figure 4 shows unary adsorption isotherms of hexane, benzene, and cyclohexane at 303 K in three materials: ZIF-8, ZIF-8-7_*de novo*, and ZIF-8-7_SACRed. For comparison between the different materials, the gravimetric uptake is normalized to the mmol of adsorbate/mol ZIF basis to circumvent the differences in molar masses of 2-MeIm and BzIm linkers. The data are presented with a log scale on the horizontal axis to facilitate a clear view of the low partial pressure region. The similar particle sizes of ∼700 nm (Figure S2) of all three materials minimize other minor effects such as differences in external surface area and interparticle capillary condensation. All three materials show much more significant uptake of *n*-hexane and benzene than cyclohexane. This is consistent with a previous observation of low cyclohexane uptake on ZIF-8.^3^ Based upon the unary isotherms, separation selectivity can be expected for benzene/cyclohexane (and hexane/cyclohexane) with all three materials, whereas only moderate separation performance maybe expected for hexane/benzene.

The hexane or benzene uptakes of the two hybrid materials drop relative to ZIF-8 due to their smaller pore volumes resulting from the introduction of the bulky BzIm linkers. The ZIF-8-7_SACRed uptake is lower than that of ZIF-8-7_*de novo*, possibly due to greater clustering of less porous BzIm-rich domains within ZIF-8-7_SACRed. Our recent reports have shown the clustering of acid gas-induced defects in the ZIF-8_HSO_*x*_ material, which is the precursor for ZIF-8-7_SACRed.^49,51,52^ The replacement of these defects with BzIm linkers would thus lead to the formation of BzIm-rich domains that would have lower porosity than 2-MeIm-rich domains.^21,23^ No qualitative differences in hexane adsorption are observed among the three materials. However, the introduction of aromatic BzIm groups in the two hybrid materials enables a higher benzene uptake (than ZIF-8) at low partial pressure due to π–π affinity interactions.^53,54^

**Figure 5:**
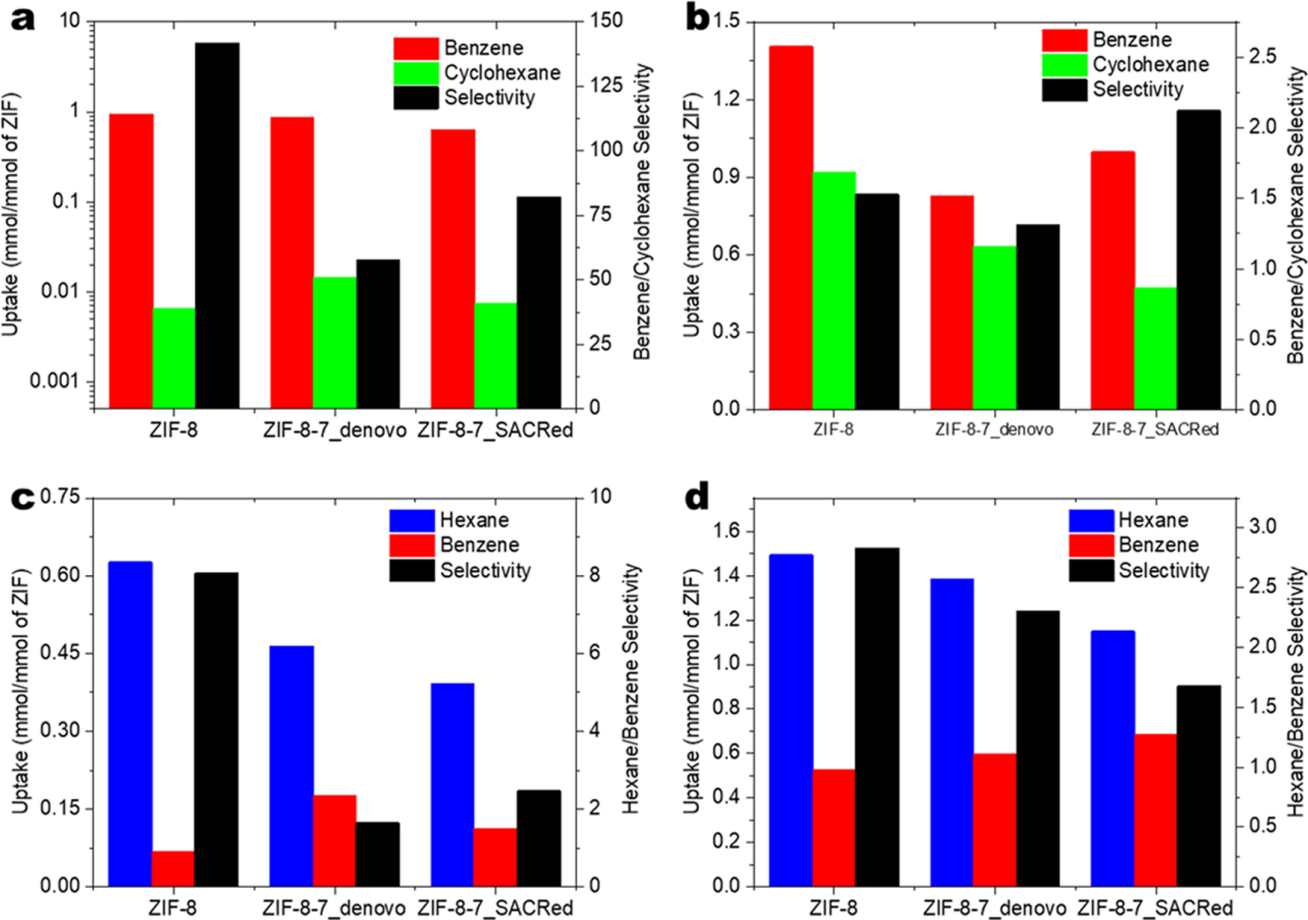
(a, c) IAST predictions and (b, d) experimental adsorption uptakes for equimolar benzene/cyclohexane and hexane/benzene mixtures, respectively.

Figure 5 compares the results of experimental liquid breakthrough adsorption measurements for hexane/benzene and benzene/cyclohexane mixtures with IAST predictions. Figure S3 shows the detailed breakthrough curves for these mixtures in all three ZIF materials, with triisopropylbenzene (TIPB) used as a nonadsorbing tracer molecule. Figure 5a shows IAST predictions (made using the unary isotherms in Figure 4) for the adsorption of an equimolar benzene/cyclohexane mixture in the three materials. IAST predicts very high benzene/cyclohexane mixture selectivity (also referred to as the separation factor and calculated from eq 2), as expected from the unary isotherms (Figure 4). However, the actual mixture behavior is significantly different (Figure 5b). While all three materials are selective for benzene over cyclohexane, the separation factors are only in the range of 1.3–2.1. While the experimentally observed benzene uptakes from the binary mixture are well predicted by IAST, the observed cyclohexane uptakes are almost two orders of magnitude larger than the IAST predictions. These observations strongly suggest that the adsorption of benzene in all the three ZIF materials creates structural changes (such as an effective increase in pore size or entropic effects related to packing arrangements) that allow coadsorption of cyclohexane. Unlike the rigid benzene molecule, cyclohexane is known to be able to take on different conformations in order to fit inside nanoporous materials, a feature that likely further increases its coadsorption in the ZIFs due to adsorption of benzene.^55^ To the best of our knowledge, this is the first definitive report of such cooperative behavior of C_6_ molecule mixtures in ZIFs, and it highlights that the rigidity of ZIF materials as observed in physisorption at 77 K does not necessarily extend to ambient temperatures and exposure to strongly adsorbing molecules.

The two ZIF-8-7 hybrid materials show different characteristics during coadsorption. Specifically, the ZIF-8-7_SACRed material has the highest benzene/cyclohexane selectivity of the three materials and shows significantly higher benzene uptake in comparison to the ZIF-8-7_*de novo* material. The adsorption of hexane/benzene mixtures provides further insight. The IAST predictions for the hexane/benzene mixture are shown in Figure 5c, whereas the experimental observations are shown in Figure 5d. IAST predicts hexane/benzene selectivities in the range of 2–8 for the three materials, based upon the greater affinity for hexane seen in Figure 4. ZIF-8 has higher hexane selectivity than the two hybrid materials, which have higher unary benzene uptakes owing to the presence of BzIm linkers. These mixture selectivity trends are generally maintained in the experimental observations of Figure 5d, albeit with lower selectivities in the range of 1.5–3. However, the experimental mixture uptakes of both hexane and benzene are about 3× higher than the IAST predictions. Once again, this indicates the effects of cooperative adsorption.

**Figure 6:**
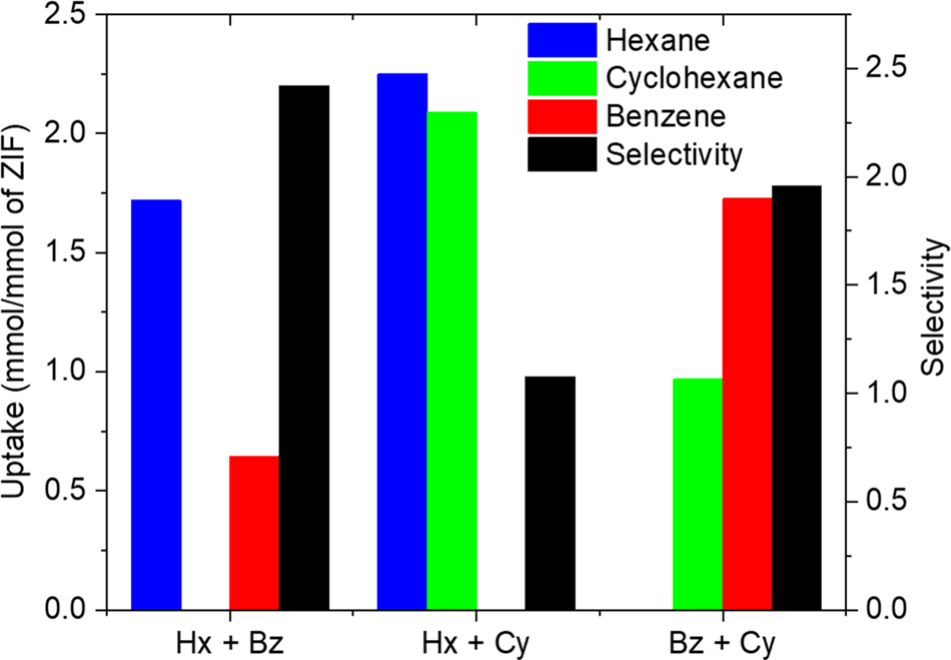
Batch adsorption of equimolar hexane/benzene, hexane/cyclohexane, benzene/cyclohexane, and pure cyclohexane (no uptake) in ZIF-8. TIPB is used as a solvent in all cases.

To further confirm these effects, batch adsorption measurements of hexane/benzene, hexane/cyclohexane, benzene/cyclohexane binary mixtures, and pure cyclohexane in ZIF-8 were performed (Figure 6). TIPB was used as a solvent in these measurements since it does not compete for adsorption within the pores. The hexane/benzene mixture uptake and selectivity in the batch measurement are consistent with the results of breakthrough measurements from Figure 5d. Similarly, no significant unary adsorption of cyclohexane was observed, consistent with the unary adsorption isotherm in Figure 4. For ZIF-8, the binary hexane/cyclohexane and benzene/cyclohexane mixtures show coadsorption of cyclohexane in the presence of either hexane or benzene, with the resulting uptakes and selectivity being consistent with the results of breakthrough measurements in Figure 5b. The batch adsorption data in Figure 6 thus confirm that the presence of strong adsorbates like hexane or benzene facilitates ambient-temperature gate-opening for the adsorption of cyclohexane. The present findings contradict earlier observations on benzene/cyclohexane separation in ZIF-8.^3^ In that work, the adsorption uptakes were measured after a multistep process of washing the adsorbate-loaded ZIF-8 crystals with *o*-xylene, drying in air flow, and dissolving in H_2_SO_4_. It is possible that these steps affected the adsorption equilibrium and led to loss of adsorbates from the ZIF-8 crystals. While the direct experimental observation of structural changes and framework dynamics in ZIFs upon hydrocarbon adsorption is difficult to accomplish due to the similarity in composition of the linkers and the adsorbates, the adsorption data provides important information on the cooperative adsorption effects.

## Conclusions

We have shown that significant structural differences exist between mixed-linker/hybrid ZIF-8-7 materials synthesized by SACRed and *de novo* methods and the corresponding effects on their adsorption and mixture separation characteristics. This work demonstrates that SACRed, a technique for introducing functional linkers into partially degraded MOFs, can generate mixed-linker hybrids with distinct physical and functional properties compared to materials synthesized by *de novo* routes. PXRD patterns and Rietveld structure refinements show that the hybrid structures retain SOD topology analogous to the parent ZIF-8. However, 2-MeIm substitution by BzIm drives symmetry reduction from *I*23 to *P*23 in ZIF-8-7_*de novo* and likely to P2 in ZIF-8-7_SACRed. The framework of ZIF-8-7_SACRed appears more distorted from the parent ZIF-8 structure than ZIF-8-7_*de novo* with nominally similar linker composition. Further, N_2_ physisorption shows significant differences in accessible pore volume and surface area between the two types of hybrid materials as well as differences in gate-opening behavior at 77 K. These structural differences, more specifically the distribution of non-native benzimidazole linker within the crystal, show a significant effect on the functional performance and the flexibility of ZIF pores. Finally, we showed that strong cooperative adsorption effects are in operation for C_6_ hydrocarbon mixtures in all three ZIF materials studied. Nevertheless, ZIF-8-7_SACRed demonstrated enhanced benzene/cyclohexane binary separation selectivity over ZIF-8 and ZIF-8-7_*de novo*, likely due to the enhanced affinity of benzene toward BzIm linkers.

## Supporting Information Available

Textural properties of ZIF materials (Table S1); solution data of ZIF-8-7 hybrids (Figure S1); SEM images of ZIF crystals (Figure S2); liquid breakthrough adsorption measurement data from ZIF materials (Figure S3) (PDF)
Numerical data for Figures 1, 4, 5, 6, S2, and S3 (XLSX)

## Notes

Notice of Copyright: This manuscript has been coauthored by UT-Battelle, LLC, under contract DE-AC05-00OR22725 with the US Department of Energy (DOE). The publisher acknowledges the US government license to provide public access under the DOE Public Access Plan (http://energy.gov/downloads/doe-public-access-plan).
