# Supplementary material for: Structural and Adsorption Properties of ZIF-8-7 Hybrid Materials Synthesized by Acid Gas-Assisted and De Novo Routes
Source: J Phys Chem C Nanomater Interfaces. 2023 Nov 30;127(49):23956–65. doi: 10.1021/acs.jpcc.3c06334 (PMC10726363; doi:10.1021/acs.jpcc.3c06334)
Supplement: Supplementary file 1 — jp3c06334_si_001.pdf [file jp3c06334_si_001.pdf]

Supporting Information

for

Structural and Adsorption Properties of ZIF-8-7 Hybrid Materials Synthesized by Acid Gas-Assisted and *de novo* Routes

Arvind Ganesan,[a] Peter C. Metz,[b] Raghuram Thyagarajan,[a] Yuchen Chang,[a] Stephen C. Purdy,[c] Krishna C. Jayachandrababu,[a] Katharine Page,[b,c] David S. Sholl,[a,d]* and Sankar Nair[a]*

[a]School of Chemical & Biomolecular Engineering, Georgia Institute of Technology, Atlanta, GA 30332, USA

[b] Materials Science and Engineering Department, University of Tennessee, Knoxville, TN 37996, USA

[c] Neutron Scattering Division, Oak Ridge National Laboratory, Oak Ridge, TN 37830, USA

[d]Oak Ridge National Laboratory, Oak Ridge, TN 37830, USA

* Corresponding author: sankar.nair@chbe.gatech.edu; shollds@ornl.gov

Keywords: MOFs, mixed-linker, structure, adsorption, hydrocarbons

Supporting Tables

Table S1. Textural characteristics of ZIF-8, ZIF-8_HSO_x, ZIF-8-7_SACRed, and ZIF-8-7_*de novo*

Sample	BET Surface Area (m^2/g)	Pore Volume (cm^3/g)
ZIF-8	1790	0.65
ZIF-8_HSO_x	993	0.35
ZIF-8-7_SACRed	1152	0.39
ZIF-8-7_*de novo*	1205	0.40

Supporting Figures

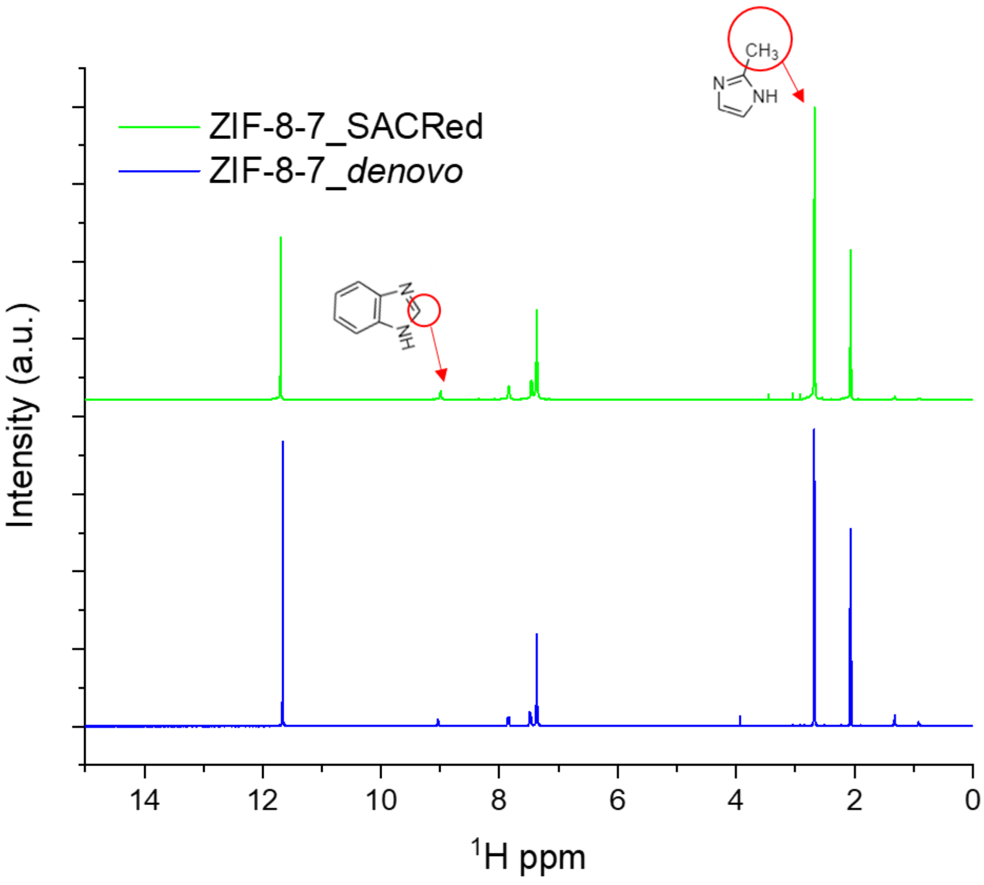

Figure S1. Solution ^{1}H NMR chemical shift spectra of ZIF-8-7 hybrids.

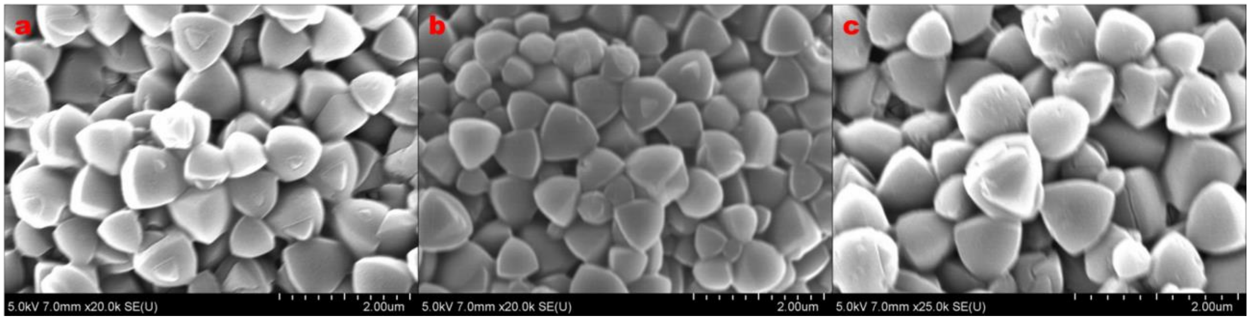

Figure S2. SEM images of **(a)** ZIF-8, **(b)** ZIF-8-7_*de novo*, and **(c)** ZIF-8-7_SACRed. All three images have the same scale bar.

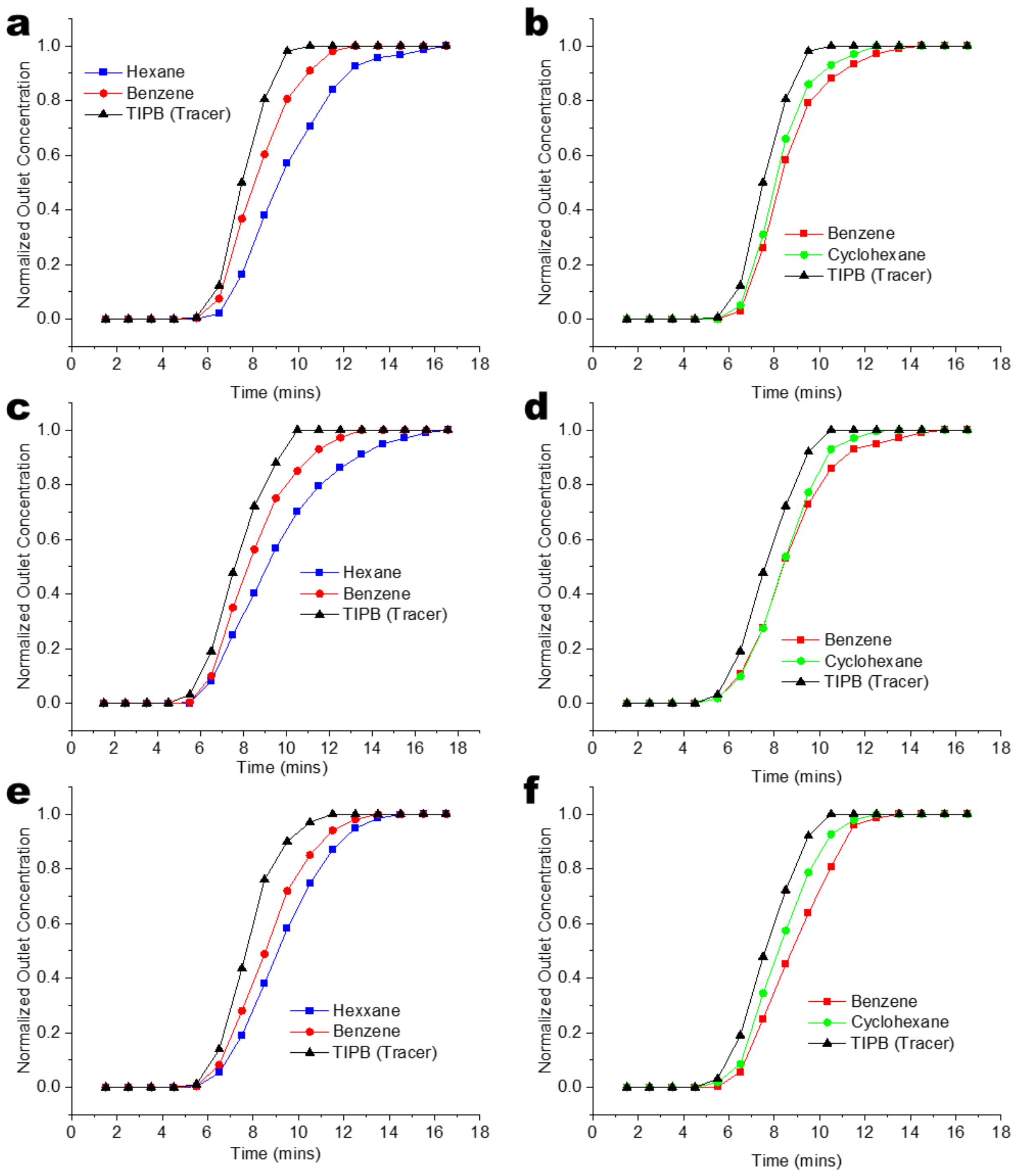

Figure S3. Liquid breakthrough measurements for binary hexane/benzene and benzene/cyclohexane mixtures respectively. **(a-b)** ZIF-8, **(c-d)** ZIF-8-7_*de novo*, and **(e-f)** ZIF-8-7_SACRed. Triisopropylbenzene (TIPB) is used as a non-adsorbing tracer.
